# Unveiling Informational Properties of the Chen-Ouillon-Sornette Seismo-Electrical Model

**DOI:** 10.3390/e23030337

**Published:** 2021-03-12

**Authors:** Hong-Jia Chen, Luciano Telesca, Michele Lovallo, Chien-Chih Chen

**Affiliations:** 1 Department of Earth Sciences, National Central University, Taoyuan 32001, Taiwan; chienchih.chen@g.ncu.edu.tw; 2 Institute of Methodologies for Environmental Analysis, National Research Council, 85050 Tito (PZ), Italy; luciano.telesca@imaa.cnr.it; 3 Agenzia Regionale per la Protezione dell’ Ambiente di Basilicata (ARPAB), 85100 Potenza, Italy; michele.lovallo@alice.it; 4 Earthquake-Disaster & Risk Evaluation and Management, National Central University, Taoyuan 32001, Taiwan

**Keywords:** Fisher information, Shannon entropy, seismo-electrical model, spring-block model, RLC circuit

## Abstract

The seismo-electrical coupling is critical to understand the mechanism of geoelectrical precursors to earthquakes. A novel seismo-electrical model, called Chen–Ouillon–Sornette (COS) model, has been developed by combining the Burridge–Knopoff spring-block system with the mechanisms of stress-activated charge carriers (i.e., electrons and holes) and pressure-stimulated currents. Such a model, thus, can simulate fracture-induced electrical signals at a laboratory scale or earthquake-related geoelectrical signals at a geological scale. In this study, by using information measures of time series analysis, we attempt to understand the influence of diverse electrical conditions on the characteristics of the simulated electrical signals with the COS model. We employ the Fisher–Shannon method to investigate the temporal dynamics of the COS model. The result showed that the electrical parameters of the COS model, particularly for the capacitance and inductance, affect the levels of the order/disorder in the electrical time series. Compared to the field observations, we infer that the underground electrical condition has become larger capacitance or smaller inductance in seismogenic processes. Accordingly, this study may provide a better understanding of the mechanical–electrical coupling of the earth’s crust.

## 1. Introduction

Earthquake prediction and forecasting has been vigorously debated; so far, scientists have developed no practical methodology [1,2,3,4,5,6]. Nevertheless, numerous research groups have reported a variety of earthquake precursors in different times and places. Such precursors include radon emissions [7,8], hydrological changes [9,10], electromagnetic signals [4,5,6,11,12,13]. Particularly, electromagnetic phenomena before earthquakes are the most promising candidate [14]. Hence, understanding precursory indicators and their generation mechanisms of preseismic electromagnetic phenomena is critical, as it may help to achieve short-term earthquake forecasts [15,16,17,18,19,20,21,22,23].

At present, the precursory mechanisms are still argumentative owing to undecided seismo-electromagnetic theories, inconsistent precursory phenomena, and shortage of objectively testable models [24,25,26,27]. Despite these reasons, scientists have significantly observed several electromagnetic anomalies within a few days or weeks before large seismic events [28,29,30,31,32] and proposed various relevant mechanisms, including solid-state physics, piezoelectric effects, electrokinetic effects, contact electrification [33,34,35,36,37,38,39,40,41,42,43,44,45]. Modeling earthquake-related geoelectrical signals, however, is still very tough, particularly for preseismic ones. Certain models [42,43,44,45] have solely simulated co-seismic electromagnetic phenomena rather than preseismic electromagnetic signals.

Recently, Chen et al. [46] have developed a seismo-electrical model to simulate electrical signals regarding rock fracturing and frictional sliding. This model combines a spring-block system [47,48,49,50,51] with the concepts of stress-activated charge carriers (i.e., electrons and holes) [34,35,36] and pressure-stimulated currents [39,40,52,53,54]. The spring-block system, originally proposed by Burridge and Knopoff in 1967 [47], can simulate stick-slip events and reproduce power-law frequency-size distributions [55,56] in a single fault or rupture zones [49,51,57]. On the other hand, the mechanisms of the stress-activated charge carriers and the pressure-stimulated currents can well justify the generation and transportation of electrical charges in seismogenic processes. As for pressure-stimulated currents, Varotsos et al. [58] also provided a useful review of a pressure-stimulated currents model proposed in the 1980s and explained that this model is compatible with experimental results deduced recently by independent research groups. After the combination, the seismo-electrical model, named Chen–Ouillon–Sornette (COS), can thus mimic fracture-induced electrical signals at a laboratory scale [40,59,60] and earthquake-related geoelectrical signals at a geological scale [18,32]. It also reproduces polar-like electromagnetic pulses that are usually observed before earthquakes [61,62,63,64,65].

Despite the self-consistent COS model, its simulated electrical signals have still uncovered properties. In this study, we simulate electrical signals through the COS model under different electrical conditions and analyze their informational properties by using the Fisher–Shannon (FS) method. In this way, we can investigate the impact of the electrical parameters of the COS model on the simulated signals. Comparing the results obtained here to the field observations, we suggest a possible evolution of underground electrical conditions during a seismogenic process. Figuring out the characteristics of the temporal organizations and structures in such simulated signals may help us to deduce the features of geoelectrical signals in real situations.

## 2. COS Seismo-Electrical Model

Within a coupled mechanical–electrical system, Chen et al. [46] have developed a fully self-consistent COS model that combines the generation of ruptures within a Burridge–Knopoff spring-block model [47,48,49,50] with the nucleation and propagation of electric pulses within an RLC-type circuit. The COS model has a theoretical framework for simulating and analyzing earthquake-related geoelectrical signals and successfully reproduces unipolar-like pulses that often precede large seismic events [61,62,63,64,65]. Moreover, it sheds some light on preseismic electromagnetic phenomena, such as variations of statistical moments [12,13,32] and transitions of power-law exponents in power spectra [19,66,67].

**Figure 1:**
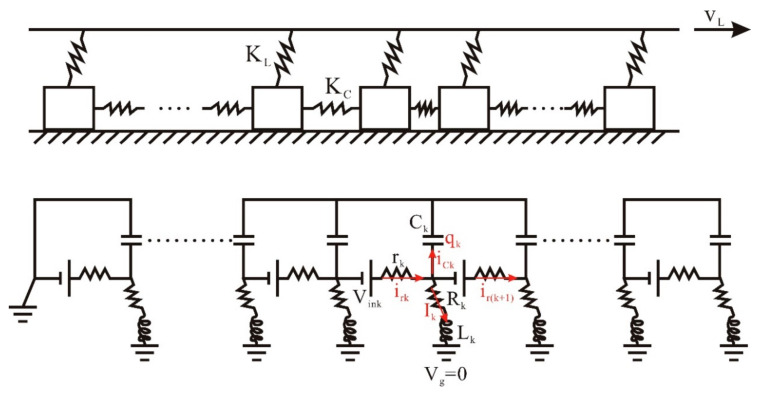
Schematic diagram of the Chen–Ouillon–Sornette (COS) seismo-electrical model (originated from Chen et al. [46]. For the spring-block system, $v_{L}$ is the velocity of the loading plate; $K_{C}$ and $K_{L}$ are the spring stiffness. For the RLC-type circuit, *r* and *c* are the block resistance and capacitance, respectively; *R* and *L* are the earth resistance and inductance surrounding the blocks, respectively; *q* is the stored electrical charge in one block; $i_{r}$, $i_{c}$, and *I* are the currents; $V_{in}$ is the stress-induced voltage. Subscript *k* means the index of the blocks. $V_{g}$ is the grounding voltage and set to be 0 V by convention.

Let us start with a one-dimensional COS model [46,48], as illustrated in Figure 1. For its mechanical part, we consider that a loading plate pulls a linear chain on a rough surface at a velocity $v_{L}$. This chain has *N* blocks of identical mass *m*; in the meantime, a spring with stiffness $K_{L}$ links the loading plate to each block, and a spring with stiffness $K_{C}$ links the adjacent blocks. Through the rough surface, all blocks are subject to friction forces. Hence, the static stability condition on the *k*-th block gives the following equation:

(1) $$K_{L}x_{k}+K_{C}\left(2x_{k}-x_{k-1}-x_{k+1}\right)=f_{rk}<f_{sk},\;\;\;\;\;\;k=1\;to\;N,$$

where $f_{rk}$ is the resulting spring force, $f_{sk}$ is the maximum static friction force between the *k*-th block and the surface, and $x_{k}$ is the position of the *k*-th block relative to the loading plate. During strain accumulation caused by the loading plate motion, all blocks are unmoving relative to the surface and have the same increment of position relative to the loading plate, which can be shown as:

(2) $$\frac{dx_{k}}{dt}=v_{L},\;\;\;\;\;\;k=1\;to\;N.$$

When the resulting spring force on the *k*-th block exceeds its maximum static friction, the block starts to slide. Its governing equation of the dynamic sliding gives the following equation:

(3) $$m\frac{d^{2}x_{k}}{dt^{2}}+K_{L}x_{k}+K_{C}\left(2x_{k}-x_{k-1}-x_{k+1}\right)=f_{dk},\;\;\;\;k=1\;to\;N,$$

where $f_{dk}$ is the dynamic friction force acting on the *k*-th block, satisfying $f_{dk}<f_{sk}$. The moving of one block may destabilize the other blocks, thus forming a multiblock sliding event. Suppose that the velocity of a moving block is nonzero or the resulting force of this block still exceeds its maximum static friction, then this block continues to slide according to Equation (3); otherwise, this block sticks to the surface owing to Equation (1).

Regarding the mechanical–electrical relationship in crustal materials, scientists have reported that the rock voltage is related to the stress gradient and a stressed rock can serve as a battery [35,68,69,70,71]. Hence, the stress-induced voltage in a rock is a function of stress, coupling the mechanical part with the electrical part in the crustal system. Here, we simply defined the stress-induced voltage on the *k*-th block as follows:

(4) $$V_{ink}(\tau_{k})=P_{dk}\beta_{k}\tau_{k}\;and\;p_{dk}=\left\{\begin{array}{c} 1\\ -1 \end{array}\begin{array}{c} ,\;\;\;\;if\;\tau_{k-1}\geq \tau_{k+1}\\ ,\;\;\;if\;\;\tau_{k-1}\leq \tau_{k+1} \end{array}\right.,$$

where $V_{ink}$ is the stress-induced voltage; $\tau_{k}$ is the stress, equivalent to the resulting spring force in Equation (1); $\beta_{k}$ is a conversion constant to convert stress to voltage [35,36,72]; $p_{dk}$ is the polarization direction of the *k*-th block, meaning that positive holes flow from higher stressed regions to lower stressed ones [70]; $p_{dk}$ is randomly assigned to ±1 when $\tau_{k-1}\;=\;\tau_{k+1}$.

As for the model’s electrical part, each block is characterized by a resistor with resistance *r* and a capacitor with capacitance *c*, which are affected by electrolyte concentrations, water content, porosity, and so on [73,74,75,76,77]. Depending on the applied stresses, the block capacitor can store or release electricity. Additionally, each block is electrically grounded because of its embedment in the earth’s crust; hence, the grounded current *I* passes through a grounded resistor with resistance *R* and a grounded inductor with inductance *L*. The grounded resistance plays an ambient resistance to the blocks, and the grounded inductance relates with the permeability of rock minerals and the ability to transform magnetic energies through electrical currents.

According to the above-mentioned architecture, we can derive the governing equations of the electrical part in the crust within an RLC-type circuit (Figure 1). First of all, Kirchhoff’s voltage law on the *k*-th block gives the following:

(5) $$\left\{\begin{array}{c} V_{in1}-i_{r1}r_{1}-\frac{q_{1}}{c_{1}}=0\\ V_{ink}-i_{rk}r_{k}-\frac{q_{k}}{c_{k}}+\frac{q_{k-1}}{c_{k-1}}=0,\;\;\;\;\;\;k=2\;to\;N^{\prime } \end{array}\right.$$

where $r_{k}$ and $c_{k}$ are the block resistance and capacitance, respectively; $i_{rk}$ is the current passing through the block resistor; $q_{k}$ is the charge stored in the block. Secondly, the current–charge relation on the *k*-th block gives the following:

(6) $$i_{ck}=\frac{dq_{k}}{dt},\;\;\;\;\;\;k=1\;to\;N,$$

where $i_{ck}$ is the current passing through the block capacitor. Thirdly, Kirchhoff’s law for the current flowing towards the neighboring blocks and the ground gives the following:

(7) $$\left\{\begin{array}{c} i_{rk}=I_{k}+i_{ck}+i_{r\left(k+1\right)},\;\;\;\;\;\;k=1\;to\;N-1\\ i_{rN}=I_{N}+i_{cN} \end{array}\right.,$$

where $I_{k}$ is the current flowing toward the ground. Finally, the voltage balance on the *k*-th block concerned with the earth terminal (i.e., the RL component) gives the following:

(8) $$\left\{\begin{array}{c} I_{1}R_{1}+\frac{dI_{1}}{dt}L_{1}=V_{in1}-i_{r1}r_{1}\\ I_{k}R_{k}+\frac{dI_{k}}{dt}L_{k}-I_{k-1}R_{k-1}-\frac{dI_{k-1}}{dt}L_{k-1}=V_{ink}-i_{rk}r_{k},\;\;\;\;\;\;k=2\;to\;N\prime \prime \end{array}\right.$$

where $R_{k}$ and $L_{k}$ are the grounded resistance and inductance, respectively. Besides, the resulting voltage of the mechanical–electrical coupling system was written as:

(9) $$V_{SB}=\frac{1}{N}\sum_{k=1}^{N}\left(R_{k}I_{k}+L_{k}\frac{dI_{k}}{dt}\right)=\frac{1}{N}\sum_{k=1}^{N}\frac{q_{k}}{c_{k}}.$$

Such a simulated voltage $V_{SB}$ can be analog to self-potential signals measured in real fields (e.g., in a fault zone). In other words, the equations simulate fractured-induced electrical signals or earthquake-related geoelectrical signals.

For convenience’ sake, the above equations are nondimensionalized by introducing the suitable variables as follows:

(10) $$T_{f}=t\sqrt{\frac{K_{L}}{m}},T_{s}=\frac{tK_{L}v_{L}}{F_{S}^{ref}},X_{i}=\frac{K_{L}x_{i}}{F_{S}^{ref}},\phi =\frac{F_{Si}}{F_{Di}},s=\frac{K_{C}}{K_{L}},\mu_{i}=\frac{F_{Si}}{F_{S}^{ref}},$$

(11) $$T=\frac{t}{c_{ref}R},\;\hat{r}=\frac{r}{R},\;\hat{c}=\frac{c}{c_{ref}},\;\hat{L}=\frac{L}{c_{ref}R^{2}},\;\hat{V_{in}}=\frac{V_{in}}{i_{ref}R},\;\hat{q}=\frac{q}{i_{ref}c_{ref}R},\;\hat{i_{r}}=\frac{i_{r}}{i_{ref}}.\;\hat{i_{c}}=\frac{i_{c}}{i_{ref}},\;\hat{I}=\frac{I}{i_{ref}}.$$

The variables in Equations (10) and (11) are used for the mechanical and electrical parts of the COS model, respectively. Hence, the equations of the mechanical part can be written as:

(12) $$X_{k}+s\left(2X_{k}-X_{k-1}-X_{k+1}\right)=\tau_{k}<\mu_{k},\;\;\;\;\;\;k=1\;to\;N,$$

(13) $$\frac{dX_{k}}{dT_{s}}=1,\;\;\;\;\;\;k=1\;to\;N,$$

(14) $$\frac{d^{2}X_{k}}{dT_{f}^{2}}+X_{k}+s\left(2X_{k}-X_{k-1}-X_{k+1}\right)=\frac{\mu_{k}}{\phi },\;\;\;\;\;k=1\;to\;N.$$

On the other hand, the equations of the electrical part can be described as:

(15) $$\hat{V_{ink}}\left(\tau_{k}\right)=p_{dk}\hat{\beta_{k}}\tau_{k},\;\;\;\;\;\;k=1\;to\;N,$$

(16) $$\left\{\begin{array}{c} \hat{V_{in1}}-\hat{i_{r1}}\hat{r_{1}}-\frac{\hat{q_{1}}}{\hat{c_{1}}}=0\\ \hat{V_{ink}}-\hat{i_{rk}}\hat{r_{k}}-\frac{\hat{q_{k}}}{\hat{c_{k}}}+\frac{\hat{q_{k-1}}}{\hat{c_{k-1}}}=0,\;\;\;\;\;\;k=2\;to\;N^{\prime } \end{array}\right.$$

(17) $$\hat{i_{ck}}=\frac{d\hat{q_{k}}}{dT},\;\;\;\;\;\;k=1\;to\;N,$$

(18) $$\left\{\begin{array}{c} \hat{i_{rk}}=\hat{I_{k}}+\hat{i_{ck}}+\hat{i_{r\left(k+1\right)}},\;\;\;\;\;\;k=1\;to\;N-1\\ \hat{i_{rN}}=\hat{I_{N}}+\hat{i_{cN}} \end{array}\right.,$$

(19) $$\left\{\begin{array}{c} \hat{I_{1}}+\frac{d\hat{l_{1}}}{dT}\hat{L_{1}}=\hat{V_{in1}}-\hat{i_{r1}}\hat{r_{1}}\\ \hat{I_{k}}+\frac{d\hat{l_{k}}}{dT}\hat{L_{k}}-\alpha_{k-1}\hat{I_{k-1}}-\alpha_{k-1}\frac{d\hat{l_{k-1}}}{dT}\hat{L_{k-1}}=\hat{V_{ink}}-\hat{i_{rk}}\hat{r_{k}},\;\;\;k\;=\;2\;to\;N^{\prime } \end{array}\right.$$

(20) $$\hat{V_{SB}}=\frac{1}{N}\sum_{k=1}^{N}\left(\hat{I_{k}}+\hat{L_{k}}\frac{d\hat{I_{k}}}{dT}\right)=\frac{1}{N}\sum_{k=1}^{N}\frac{\hat{q_{k}}}{\hat{c_{k}}}.$$

These nondimensional equations enable us to investigate the relationships between the electrical parameters and the time series of the simulated voltages.

**Figure 2:**
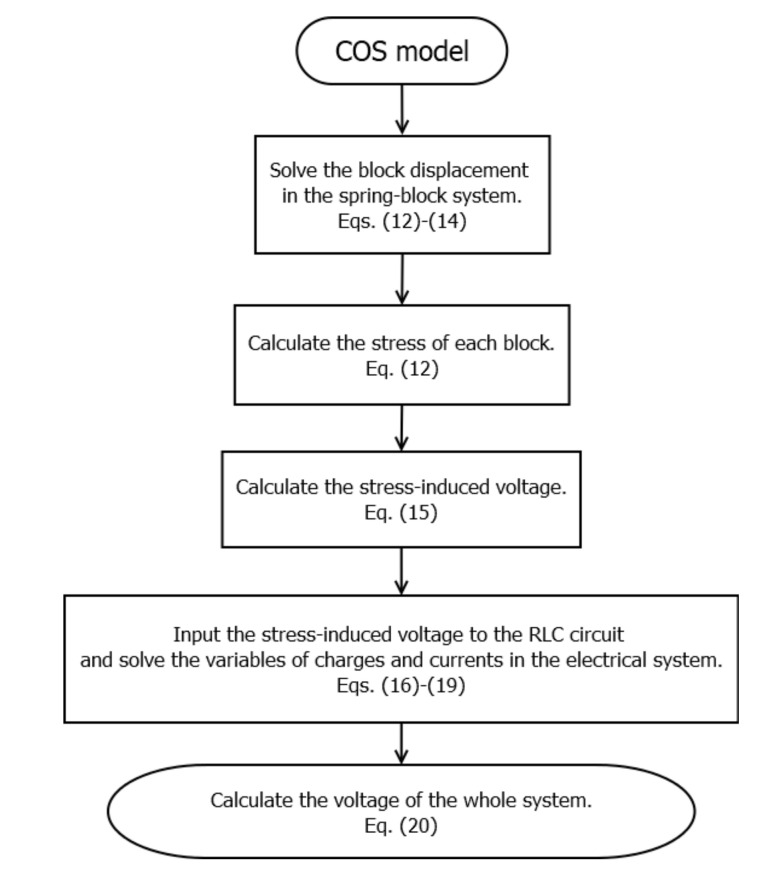
Flowchart of the COS seismo-electrical model.

To elucidate the process of how the simulated voltages are generated in the coupled mechanical–electrical system, Figure 2 shows the flowchart of the COS model. Based on Equations (12)–(14), we first solve the block displacement of the spring-block system. Subsequently, we calculate the stress of each block based on Equation (12). Then, we calculated the stress-induced voltage by using Equation (15). Given the obtained stress-induced voltage, we can solve the variables of charges and currents in Equations (16)–(19). Finally, we obtained the simulated voltages of the spring-block system through Equation (20).

**Figure 3:**
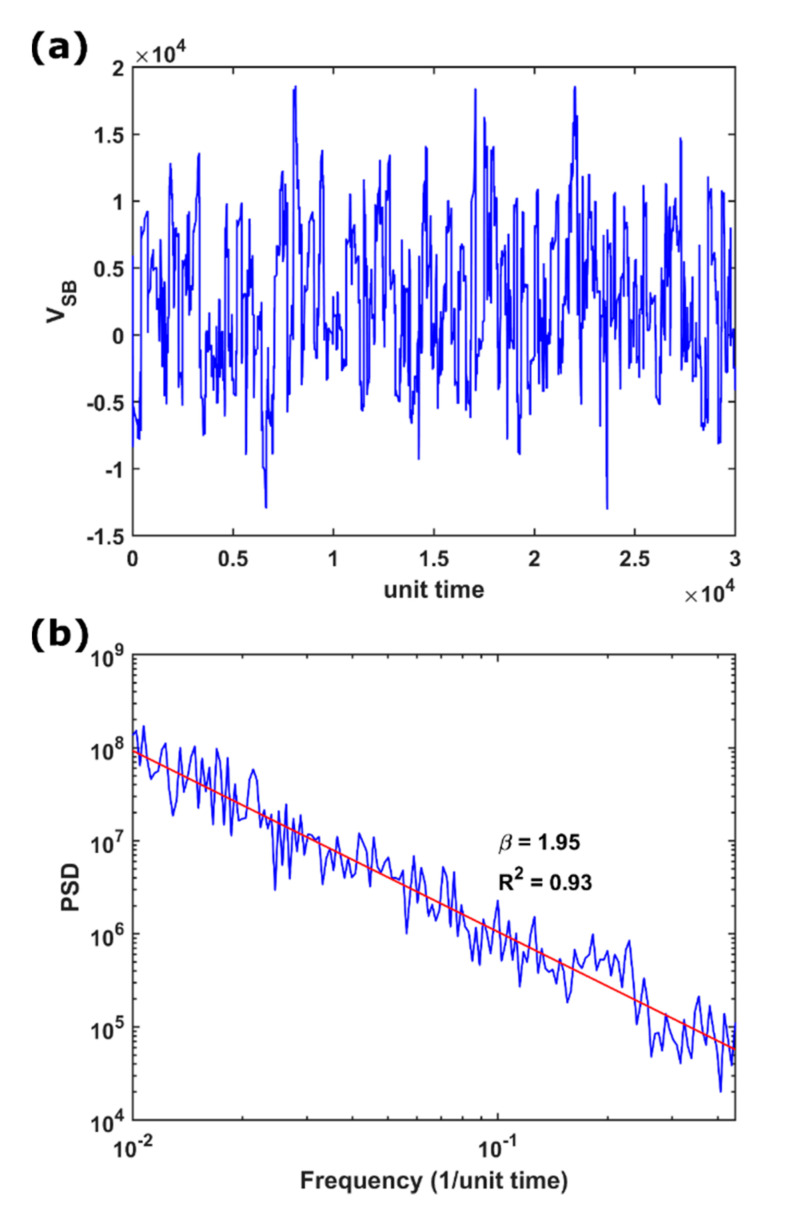
(**a**) Time series of the voltages simulated under the electrical parameters of $\hat{r}=5$, $\hat{L}=5$, and $\hat{c}=0.001$. (**b**) Power spectral density (PSD) of the time series in (**a**). The red line represents the fitting of the $1/f^{-\beta }$ noise with the scaling of β=1.95 and the R-squared $R^{2}$ of 0.93.

In this study, we set N to be 128, s to be 30, ϕ to be 1.5, and μ was randomly assigned between 1 and 3.5 for each block. In addition, we set diverse values to $\hat{r}$, $\hat{c}$, and $\hat{L}$ to investigate the influence of changing the electrical parameters on the temporal dynamics of the simulated voltages $\hat{V_{SB}}$. For instance, Figure 3a,b shows the time series and its power spectral density (PSD), respectively, of the voltages simulated under the parameters of $\hat{r}=5$, $\hat{L}=5$, and $\hat{c}=0.001$. We observed that such time series were similar to real ones (e.g., in comparison to those in Figure 2 in [78] at a laboratory scale and those in Figure 2 in [79] at a geological scale). In Figure 3b, the PSD of the simulated voltages conforms to the $1/f^{-\beta }$ noise with the power-law scaling of β=1.95; the scaling for geoelectrical signals usually ranges from 1 to 2 [66,67,79]. Therefore, the COS model can reproduce pulse-like behaviors in the time domain and the scaling behavior in the frequency domain.

## 3. FS Method

To get information regarding the temporal dynamics of the simulated voltages, we employed the well-known FS method. Fisher developed a measure, in terms of probability density functions (PDFs), to discuss the loss of data information [80]. On the other hand, Shannon introduced the concept of information entropy to data communication for investigating how well data from the source can be losslessly compressed onto a perfectly noiseless channel [81]. The FS method jointly uses the Fisher information measure and the Shannon information entropy, which are efficient statistical indices for studying the dynamics of complex nonstationary time series and the change of physical systems [82,83,84,85,86,87]. For example, the FS method is utilized for portraying the temporal evolution of physical processes, suggesting the direction of decreasing accuracy for the determination of the mean value of a physical parameter [83].

Moreover, scientists have used the FS method and studied various complex geophysical and environmental phenomena to reveal informational properties of the mechanisms governing their temporal dynamics [88,89,90,91,92] and detect precursors of catastrophic events [93,94,95,96,97]. For example, Telesca and Lovallo analyzed hourly wind speed time series at several heights above the ground level, finding that the FS informational properties of the wind data are height-dependent [90]. Furthermore, Telesca et al. applied the FS method to discriminate between tsunamigenic and nontsunamigenic earthquake seismograms, suggesting that this method may efficiently speed up the tsunami warning time [91]. Therefore, the FS method is regarded as an efficient data exploration tool.

Now, let us introduce the relevant Fisher and Shannon quantities. Assuming that $p\left(x\right)$ is the PDF of variable x, its Fisher information measure $I_{x}$ can be written as:

(21) $$I_{x}=\int_{-\infty }^{\infty }{\left(\frac{\partial }{\partial x}p\left(x\right)\right)}^{2}\frac{dx}{p\left(x\right)},$$

and its Shannon entropy $H_{x}$ can be described as:

(22) $$H_{x}=-\int_{-\infty }^{\infty }p\left(x\right)log\left[p\left(x\right)\right]dx.$$

Comparing Equation (21) with Equation (22), the major difference between the integrands is a squared derivative of $p\left(x\right)$. Thus, the Fisher information offers a local measure of the concentration of the PDFs, whereas the Shannon entropy gives a global measure [83,84]. The Shannon entropy is nonnegative for discrete distributions and can take any real positive and negative values for continuous distributions. To avoid the difficulty arising with negative information measures, we used the Shannon entropy power $N_{x}$ instead of the entropy $H_{x}$ and obtained the following equation:

(23) $$N_{x}=\frac{1}{2\pi e}e^{2H_{x}}.$$

Applying both $I_{x}$ and $N_{x}$ satisfies the “isoperimetric inequality”, a lower bound to the FS product $I_{x}N_{x}$ of ≥D, where D is the dimension of the space of the variable x [98]. Such an isoperimetric inequality indicates that the Fisher information measure and the Shannon entropy power are intrinsically linked to each other; hence, the dynamics of complex time series can be characterized by using the two measures jointly in the FS information plane. The product $I_{x}N_{x}$ can be considered as a statistical measure of complexity [99,100]. The “isocomplexity line” $I_{x}N_{x}=1$ separates the FS plane into two parts, and the distance of a signal point to this line can quantify the degree of the signal complexity.

The estimation of the FS quantities depends on the calculation of a PDF. A rough approximation of the unknown PDF is given by the histogram. Nevertheless, we can smoothly and robustly estimate the PDF utilizing the kernel density estimator technique [101,102] that approximates $p\left(x\right)$ as:

(24) $${\hat{p}}_{M}\left(x\right)=\frac{1}{Mb}\sum_{i=1}^{M}K\left(\frac{x-x_{i}}{b}\right),$$

where b is the bandwidth and M is the number of data. The kernel function $K\left(u\right)$ is a continuous nonnegative and symmetric function satisfying the two following conditions:

(25) $$K\left(u\right)\geq 0\;and\;\;\int_{-\infty }^{\infty }K\left(u\right)du=1.$$

Here, we employed a Gaussian kernel with zero mean and unit variance [103,104], so that $p\left(x\right)$ was estimated as:

(26) $${\hat{p}}_{M}\left(x\right)=\frac{1}{M\sqrt{2\pi b^{2}}}\sum_{i=1}^{M}e^{-\frac{{\left(x-x_{i}\right)}^{2}}{b^{2}}},$$

where the bandwidth b is estimated through an optimization process, described in Telesca and Lovallo [105].

## 4. Results

**Figure 4:**
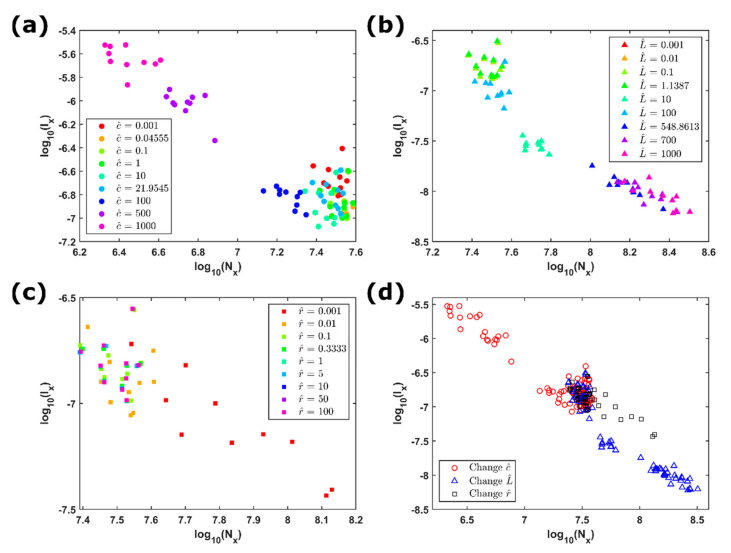
Fisher–Shannon (FS) information plane of the simulated voltages: (**a**) the voltages simulated through the COS model for $\hat{r}=5$ and $\hat{L}=5$, while increasing $\hat{c}$ from 0.001 to 1000. Each case has 10 simulations with different initial block positions; (**b**) the voltages with the fixed parameters $\hat{r}=5$ and $\hat{c}=5$ and the increase of $\hat{L}$ from 0.001 to 1000; (**c**) the voltages with the fixed parameters $\hat{c}=5$ and $\hat{L}=5$ and the increase of $\hat{r}$ from 0.001 to 100; (**d**) FS information plane for all cases from (**a**) to (**c**). Red circles represent the simulations of changing $\hat{c}$, blue triangles changing $\hat{L}$, and black squares changing $\hat{r}$.

To understand the temporal dynamics of the COS model, we investigated several time series of voltages simulated under diverse electrical conditions. Figure 4 lists the quantities of the resistance, capacitance, and inductance used in the simulation. In our strategy, we simulated 10 sets of the realizations of the voltage time series for each parameter set; each time series was simulated with random initial conditions of block positions. For each time series, we estimated its Fisher information measure $I_{x}$ and Shannon entropy power $N_{x}$. Then, we compared the behaviors of the two indicators by fixing two parameters among capacitance, resistance, and inductance and varying the remaining one.

At the beginning, we researched the effect of the capacitance on the voltage time series. Figure 4a displays the FS indices on the information plane for the voltages simulated by assigning $\hat{r}=5$ and $\hat{L}=5$ and increasing $\hat{c}$ from 0.001 to 1000. The distribution of the FS indices has a trend that the values of the two indices with a larger capacitance fall in the region of higher $I_{x}$ and lower $N_{x}$. This means that the temporal dynamics of the voltage time series is characterized by higher local order and lower global disorder with the increasing quantity of the capacitance, particularly for $\hat{c}\geq 500$. As for the effect of the inductance, Figure 4b presents the FS information plane for the voltages simulated by fixing $\hat{r}=5$ and $\hat{c}=5$ and increasing $\hat{L}$ from 0.001 to 1000. A clear pattern is visible: when the inductance increases, $N_{x}$ increases and $I_{x}$ decreases, indicating a tendency to lose the order and augment the uncertainty at both local and global scales with the increase of the inductance. In terms of the resistance, Figure 4c shows the FS information plane for the voltages simulated by fixing $\hat{c}=5$ and $\hat{L}=5$ and increasing $\hat{r}$ from 0.001 to 100. We observed a discrimination that the FS indices with the lowest resistance ($\hat{r}=0.001$) distinguish from the indices of all the other resistances and spread in the area of larger $N_{x}$. This indicated that for the lowest resistance the signals are characterized by the large uncertainty or disorder.

To sum up, Figure 4d shows the FS indices of all the simulated voltages on the same information plane. Generally speaking, three clusters can be identified. First, the voltages simulated with $\hat{c}\geq 500$ occupy mostly the area of larger $I_{x}$ and smaller $N_{x}$; second, the voltages simulated with $\hat{L}\geq 500$ occupy the area of smaller $I_{x}$ and larger $N_{x}$; third, the voltages simulated by changing $\hat{r}$ occupy the area between the two previous clusters, mixing with those of changing $\hat{c}$ smaller than 500 and of changing $\hat{L}$ smaller than 500. Those results suggested that the levels of the local and global orders of the simulated voltages mainly depend on the quantities of the capacitance and inductance rather than resistance. We note, in this study, that the FS information planes show the results with fixed values of five for any two electrical parameters in all the simulations. When changing the fixed values of the electrical parameters, the quantities $I_{x}$ and $N_{x}$ change. However, the tendencies obtained here keep preserved, i.e., the increase of the order with increasing the capacitance and the increase of the disorder with increasing the inductance.

**Figure 5:**
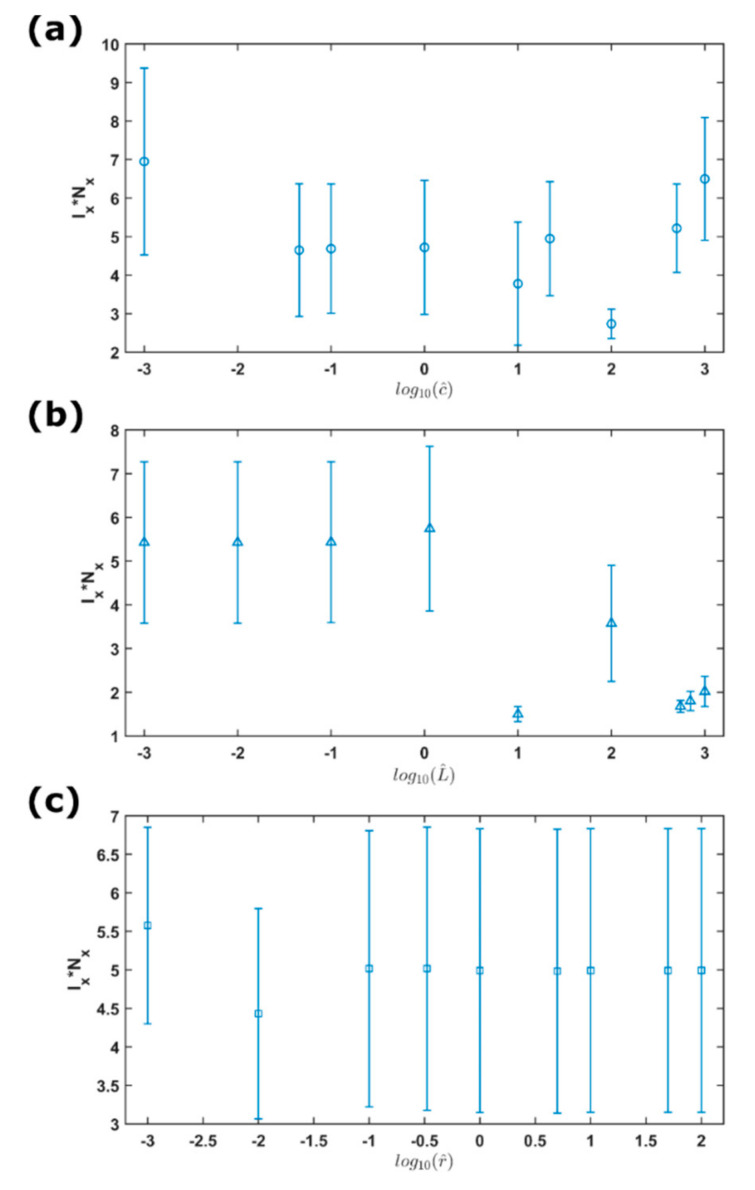
Complexity ($I_{x}N_{x}$) for the simulations of changing $\hat{c}$ (**a**), changing $\hat{L}$ (**b**), and changing $\hat{r}$ (**c**). Each vertical line represents the mean value within one standard error.

Besides, we defined a statistical measure of complexity, which can be expressed as the product of Fisher information measure and Shannon entropy power ($I_{x}N_{x}$) [100]. The statistical complexity represents a combined effect of both local and global factors, offering an evaluation of the organization, structure, and correlation in a system or time series. Figure 5 shows the complexity versus the capacitance (Figure 5a), inductance (Figure 5b), and resistance (Figure 5c). All the results showed that $I_{x}N_{x}>1$. First, we observed that the complexity versus the capacitance shows the lowest value of around 3 when $\hat{c}=100$. As for the inductance, the complexity exhibits two regimes separated by $\hat{L}=1$, showing that the mean value is around 5.5 when $\hat{L}\leq 1$ whereas that is mostly 2 when $\hat{L}>1$. In terms of the resistance, the complexity changes insignificantly with $\hat{r}$, most of which show a mean value of five.

## 5. Discussion and Conclusions

In this study, we investigated the FS information of the voltages simulated through the diverse electrical conditions of the COS seismo-electrical model. According to the simulation results, the quantities of the capacitance and inductance affect the levels of the local and global orders of the simulated voltages, while the changes of the resistance mostly do not affect their informational properties. In this section, we discussed the dependence of the electrical conditions on the organization and structure of the simulated voltages.

Establishing the seismo-electrical model, Chen et al. [46] have derived its analytical solutions for the single-block architecture, showing that the three electrical parameters (resistance, capacitance, and inductance) impact on the waveform of the charge Green function (see Equations (19)–(21) and Figure 2 in [46]). According to the different quantities of the electrical parameters, one sliding event generates the diversity of voltage time series. The generated voltages are featured by two predominant factors: decay time and amplitude. The decay time is controlled by the resistance, capacitance, and inductance, while the amplitude is dominated by the resistance and is reversely proportional to the resistance. The voltage time series generated by the present sliding event is fluctuated with the voltages generated by the past sliding events. Particularly when the decay time of the voltage time series is longer than the interevent time of two consecutive sliding events, the resulting voltage time series become more disordered. On the other hand, as mentioned above, the small amplitudes of the voltages can be due to the large resistance. Such small amplitudes lead to a concentrated distribution of its voltage time series. This concentrated distribution results in large $I_{x}$ and small $N_{x}$, indicating an ordered state [87]. Even if the decay time is longer than the interevent time, the voltage time series show less disorder owing to the superposition of small amplitudes of the voltages generated by the sliding events. Hence, the dynamics (the state of disorder) of the simulated voltages is seriously affected by the electrical parameters owing to the length of the decay time and the amplitude of the voltage.

From the perspective of physics, the capacitance C of a capacitor is the ratio of the magnitude of the stored charge Q to the magnitude of the voltage V across the capacitor:

(27) $$C=\frac{Q}{V}\;or\;V=\frac{Q}{C}.$$

We rewrote Equation (27) in a differential form:

(28) $$\frac{dQ}{dt}=C\frac{dV}{dt}.$$

Based on Equation (28), we understand that the capacitance tends to resist the change of voltages. Given a small change of the electric charges, increasing the capacitance reduces the change of the voltages. Hence, the voltages simulated with larger capacitance show the localization of the time series, leading to the state of order (i.e., higher $I_{x}$ and lower $N_{x}$). On the other hand, the voltage across an inductor of inductance L is proportional to the time rate of change of the current flowing through it, as follows:

(29) $$V=L\frac{dI}{dt}.$$

We observed the form of Equation (29) and figured out that the inductance tends to amplify voltage changes. When the rate of current change dI/dt is subject to a slight fluctuation in time, a larger inductance leads to a larger voltage variation. Therefore, increasing the inductance of the COS model exhibits a scattered distribution of the simulated voltages, leading to a disordered state of the time series (i.e., lower $I_{x}$ and higher $N_{x}$). Understanding the effects of the electrical parameters helps us to identify the electrical signals in real situations. As a result, the COS model contributes to a deeper understanding of the mechanical–electrical coupling of the crustal system.

In field observations, several studies have applied the FS method to investigate the relationship between geoelectrical signals and seismic events [93,94,95,96,97]. For instance, Potirakis et al. investigated the electromagnetic signals before, during, and after strong earthquakes through the FS method [96]. They found that the higher level of the order in the electromagnetic signals precedes the earthquake occurrences, indicating that the FS method reliably distinguishes the candidate electromagnetic precursors from noises. The geoelectrical signals preceding the seismic events seem to exhibit higher organized and ordered states, which can be simulated with larger capacitance or smaller inductance in the COS model. Combining the field observations with the results in this study, we reasonably infer that the electrical properties of the earth’s crust have become larger capacitance or smaller inductance in the seismogenic processes. Several possible explanations may be provided for such a variation. The electrical characteristics of the crust are affected by rock composition, porosity, fluid permeability, permittivity, and so forth [17,33,44]. For instance, during an earthquake preparation, dielectric crystals may be polarized towards a preferred orientation under tectonic stresses [17,58]; hence, the permittivity increases. In electromagnetism, the capacitance is proportional to the permittivity. This suggests that highly organized electrical structures due to seismogenic processes generate highly ordered geoelectrical signals with larger Fisher information and smaller Shannon entropy.

In conclusion, the results obtained in this study might not only gain insights into a better understanding of the complexity of the mechanical–electrical mechanisms in the earth’s crust but also be useful for developing the detection of preseismic electromagnetic signals. Despite this, some possibilities remain for future work that follows the present study. The COS model used is currently a one-dimension case and can be further developed into a two-dimension architecture. In this way, the characteristics of the simulated voltages are expected to approach those of real geoelectrical signals. Moreover, besides the information measures, it is worth exploring more properties of the COS model through other methodologies, for example, natural time analysis [106,107,108,109,110]. The results of such studies will be reported elsewhere.

## Data Availability

The data that support the findings of this study are openly available in Mendeley Data at http://dx.doi.org/10.17632/x9k4ypjrmj.1. We accessed the data on 27 August 2020.

## References

[1] Jordan T.H., Chen Y.-T., Gasparini P., Madariaga R., Main I., Marzocchi W., Papadopoulos G., Sobolev G., Yamaoka K., Zschau J. (2011). Operational Earthquake Forecasting: State of Knowledge and Guidelines for Utilization. Ann. Geophys..

[2] Wyss M. (2001). Why is earthquake prediction research not progressing faster?. Tectonophysics.

[3] Geller R.J. (1997). Earthquake prediction: A critical review. Geophys. J. Int..

[4] Uyeda S. (1998). VAN method of short-term earthquake prediction shows promise. Eos Trans. Am. Geophys. Union.

[5] Varotsos P., Eftaxias K., Vallianatos F., Lazaridou M. (1996). Basic principles for evaluating an earthquake prediction method. Geophys. Res. Lett..

[6] Lighthill S.J. (1996). A Critical Review of Van: Earthquake Prediction from Seismic Electrical Signals.

[7] Fu C.-C., Walia V., Yang T.F., Lee L.-C., Liu T.-K., Chen C.-H., Kumar A., Lin S.-J., Lai T.-H., Wen K.-L. (2017). Preseismic anomalies in soil-gas radon associated with 2016 M 6.6 Meinong earthquake, Southern Taiwan. Terr. Atmos. Ocean. Sci..

[8] Petraki E., Nikolopoulos D., Panagiotaras D., Cantzos D., Yannakopoulos P., Nomicos C., Stonham J. (2015). Radon-222: A Po-tential Short-Term Earthquake Precursor. J. Earth Sci. Clim. Chang..

[9] Chien S.-H.J., Chi W.-C., Ke C.-C. (2020). Precursory and coseismic groundwater temperature perturbation: An example from Taiwan. J. Hydrol..

[10] Chen C.-H., Tang C.-C., Cheng K.-C., Wang C.-H., Wen S., Lin C.-H., Wen Y.-Y., Meng G., Yeh T.-K., Jan J.C. (2015). Groundwater–strain coupling before the 1999 M w 7.6 Taiwan Chi-Chi earthquake. J. Hydrol..

[11] Hattori K. (2004). ULF Geomagnetic Changes Associated with Large Earthquakes. Terr. Atmos. Ocean. Sci..

[12] Chen H.-J., Chen C.-C. (2016). Testing the correlations between anomalies of statistical indexes of the geoelectric system and earthquakes. Nat. Hazards.

[13] Chen H.-J., Chen C.-C., Ouillon G., Sornette D. (2017). Using Skewness and Kurtosis of Geoelectric Fields to Forecast the 2016/2/6, ML6.6 Meinong, Taiwan Earthquake. Terr. Atmos. Ocean. Sci..

[14] Hayakawa M., Sandhu A., Okada H. (2016). Earthquake Prediction with Electromagnetic Phenomena. AIP Conf. Proc..

[15] Morgounov V., Malzev S. (2007). A multiple fracture model of pre-seismic electromagnetic phenomena. Tectonophysics.

[16] Eftaxias K., Contoyiannis Y., Balasis G., Karamanos K., Kopanas J., Antonopoulos G., Koulouras G., Nomicos C. (2008). Evidence of fractional-Brownian-motion-type asperity model for earthquake generation in candidate pre-seismic electromagnetic emissions. Nat. Hazards Earth Syst. Sci..

[17] Huang Q. (2011). Rethinking earthquake-related DC-ULF electromagnetic phenomena: Towards a physics-based approach. Nat. Hazards Earth Syst. Sci..

[18] Petraki E., Nikolopoulos D., Nomicos C., Stonham J., Cantzos D., Yannakopoulos P., Kottou S. (2015). Electromagnetic Pre-earthquake Precursors: Mechanisms, Data and Models-A Review. J. Earth Sci. Clim. Chang..

[19] Eftaxias K., Kapiris P., Polygiannakis J., Peratzakis A., Kopanas J., Antonopoulos G., Rigas D. (2003). Experience of short term earthquake precursors with VLF–VHF electromagnetic emissions. Nat. Hazards Earth Syst. Sci..

[20] Hayakawa M., Hobara Y. (2010). Current status of seismo-electromagnetics for short-term earthquake prediction. Geomat. Nat. Hazards Risk.

[21] Schekotov A., Chebrov D., Hayakawa M., Belyaev G., Berseneva N. (2020). Short-term earthquake prediction in Kamchatka using low-frequency magnetic fields. Nat. Hazards.

[22] Varotsos P.A., Sarlis N.V., Skordas E.S., Lazaridou M.S. (2013). Seismic Electric Signals: An additional fact showing their physical interconnection with seismicity. Tectonophysics.

[23] Varotsos P.A., Sarlis N.V., Skordas E.S. (2003). Long-range correlations in the electric signals that precede rupture: Further investigations. Phys. Rev. E.

[24] Tzanis A., Vallianatos F., Gruszow S. (2000). Identification and discrimination of transient electrical earthquake precursors: Fact, fiction and some possibilities. Phys. Earth Planet. Inter..

[25] Eftaxias K., Kapiris P., Polygiannakis J., Bogris N., Kopanas J., Antonopoulos G., Peratzakis A., Hadjicontis V. (2001). Signature of pending earthquake from electromagnetic anomalies. Geophys. Res. Lett..

[26] Pham V.N., Geller R.J. (2002). Comment on “Signature of pending earthquake from electromagnetic anomalies” by K. Eftaxias et al. Geophys. Res. Lett..

[27] Park S.K., Dalrymple W., Larsen J.C. (2007). The 2004 Parkfield earthquake: Test of the electromagnetic precursor hypothesis. J. Geophys. Res. Solid Earth.

[28] Uyeda S., Nagao T., Kamogawa M. (2009). Short-term earthquake prediction: Current status of seismo-electromagnetics. Tectonophysics.

[29] Chen Y.-I., Huang C.-S., Liu J.-Y. (2015). Statistical evidences of seismo-ionospheric precursors applying receiver operating characteristic (ROC) curve on the GPS total electron content in China. J. Asian Earth Sci..

[30] Han P., Hattori K., Zhuang J., Chen C.-H., Liu J.-Y., Yoshida S. (2017). Evaluation of ULF seismo-magnetic phenomena in Kakioka, Japan by using Molchan’s error diagram. Geophys. J. Int..

[31] Sarlis N.V. (2018). Statistical Significance of Earth’s Electric and Magnetic Field Variations Preceding Earthquakes in Greece and Japan Revisited. Entropy.

[32] Chen H.-J., Chen C.-C., Ouillon G., Sornette D. (2021). A paradigm for developing earthquake probability forecasts based on geoelectric data. Eur. Phys. J. Spec. Top..

[33] Eccles D., Sammonds P.R., Clint O.C. (2005). Laboratory studies of electrical potential during rock failure. Int. J. Rock Mech. Min. Sci..

[34] Freund F. (2010). Toward a unified solid state theory for pre-earthquake signals. Acta Geophys..

[35] Takeuchi A., Lau B.W.S., Freund F.T. (2006). Current and surface potential induced by stress-activated positive holes in igneous rocks. Phys. Chem. Earth Parts A B C.

[36] Takeuchi A., Nagao T. (2013). Activation of hole charge carriers and generation of electromotive force in gabbro blocks subjected to nonuniform loading. J. Geophys. Res. Solid Earth.

[37] Rabinovitch A., Frid V., Bahat D. (2007). Surface oscillations—A possible source of fracture induced electromagnetic radiation. Tectonophysics.

[38] Vallianatos F., Tzanis A. (1998). Electric current generation associated with the deformation rate of a solid: Preseismic and coseismic signals. Phys. Chem. Earth.

[39] Vallianatos F., Triantis D., Tzanis A., Anastasiadis C., Stavrakas I. (2004). Electric earthquake precursors: From laboratory results to field observations. Phys. Chem. Earth Parts A B C.

[40] Vallianatos F., Triantis D. (2008). Scaling in Pressure Stimulated Currents related with rock fracture. Phys. A Stat. Mech. Its Appl..

[41] Yoshida S., Uyeshima M., Nakatani M. (1997). Electric potential changes associated with slip failure of granite: Preseismic and coseismic signals. J. Geophys. Res. Solid Earth.

[42] Revil A., Jardani A. (2010). Seismoelectric response of heavy oil reservoirs: Theory and numerical modelling. Geophys. J. Int..

[43] Zhu Z., Toksöz M.N. (2012). Experimental measurements of the streaming potential and seismoelectric conversion in Berea sandstone. Geophys. Prospect..

[44] Huang Q., Ren H., Zhang D., Chen Y.J. (2015). Medium effect on the characteristics of the coupled seismic and electromagnetic signals. Proc. Jpn. Acad. Ser. B.

[45] Ren H., Huang Q., Chen X. (2016). Numerical simulation of seismo-electromagnetic fields associated with a fault in a porous medium. Geophys. J. Int..

[46] Chen H.-J., Chen C.-C., Ouillon G., Sornette D. (2021). Coupled mechano-electrokinetic Burridge-Knopoff model of fault sliding events and transient geoelectric signals. Eur. Phys. J. Spec. Top..

[47] Burridge R., Knopoff L. (1967). Model and Theoretical Seismicity. Bull. Seismol. Soc. Am..

[48] Abaimov S.G., Turcotte D.L., Shcherbakov R., Rundle J.B. (2007). Recurrence and interoccurrence behavior of self-organized complex phenomena. Nonlinear Process. Geophys..

[49] Brown S.R., Scholz C.H., Rundle J.B. (1991). A simplified spring-block model of earthquakes. Geophys. Res. Lett..

[50] Carlson J.M. (1991). Two-dimensional model of a fault. Phys. Rev. A.

[51] Pelletier J.D., Rundle J.B., Turcotte D.L., Klein W. (2000). Spring-Block Models of Seismicity: Review and Analysis of a Structurally Heterogeneous Model Coupled to a Viscous Asthenosphere. Geocomplexity and the Physics of Earthquakes.

[52] Varotsos P., Alexopoulos K., Nomicos K. (1982). Comments on the Pressure Variation of the Gibbs Energy for Bound and Unbound Defects. Phys. Status Solidi.

[53] Varotsos P., Alexopoulos K. (1984). Physical properties of the variations of the electric field of the earth preceding earthquakes. II. determination of epicenter and magnitude. Tectonophysics.

[54] Varotsos P., Alexopoulos K., Lazaridou M. (1993). Latest aspects of earthquake prediction in Greece based on seismic electric signals, II. Tectonophysics.

[55] Gutenberg B.A., Richter C.F. (1954). Seismicity of the Earth and Related Phenomena.

[56] Hainzl S., Zöller G., Kurths J. (1999). Similar power laws for foreshock and aftershock sequences in a spring-block model for earthquakes. J. Geophys. Res. Solid Earth.

[57] Ruff L.J. (1992). Asperity distributions and large earthquake occurrence in subduction zones. Tectonophysics.

[58] Varotsos P.A., Sarlis N.V., Skordas E.S. (2019). Phenomena preceding major earthquakes interconnected through a physical model. Ann. Geophys..

[59] Mavromatou C., Hadjicontis V., Ninos D., Mastrogiannis D., Eftaxias K., Hadjicontis E. (2004). Understanding the fracture phenomena in inhomogeneous rock samples and ionic crystals, by monitoring the electromagnetic emission during their deformation. Phys. Chem. Earth Parts A B C.

[60] Yang C., Liu S., Liu J., Yang H., Xie J. (2019). Characteristics of self-potential of coal samples under uniaxial compression. J. Appl. Geophys..

[61] Nenovski P. (2018). Underground current impulses as a possible source of unipolar magnetic pulses. Acta Geod. Geophys..

[62] Scoville J.T., Heraud J., Freund F. (2015). Pre-earthquake magnetic pulses. Nat. Hazards Earth Syst. Sci..

[63] Bleier T., Dunson C., Maniscalco M., Bryant N., Bambery R., Freund F. (2009). Investigation of ULF magnetic pulsations, air conductivity changes, and infra red signatures associated with the 30 October Alum Rock M5.4 earthquake. Nat. Hazards Earth Syst. Sci..

[64] Bleier T., Dunson C., Alvarez C., Freund F., Dahlgren R. (2010). Correlation of pre-earthquake electromagnetic signals with laboratory and field rock experiments. Nat. Hazards Earth Syst. Sci..

[65] Bleier T., Dunson C., Roth S., Heraud J., Lira A., Freund F., Dahlgren R., Bambery R., Bryant N., Liu J.Y., Hayakawa M. (2013). Ground-based and space-based electromagnetic monitoring for pre-earthquake signals. Earthquake Prediction Studies: Seismo Electromagnetics.

[66] Ramírez-Rojas A., Pavía-Miller C., Angulo-Brown F. (2004). Statistical behavior of the spectral exponent and the correlation time of electric self-potential time series associated to the Ms=7.4 14 September 1995 earthquake in Mexico. Phys. Chem. Earth Parts A B C.

[67] Petraki E., Nikolopoulos D., Chaldeos Y., Koulouras G., Nomicos C., Yannakopoulos P.H., Kottou S., Stonham J. (2016). Fractal evolution of MHz electromagnetic signals prior to earthquakes: Results collected in Greece during 2009. Geomat. Nat. Hazards Risk.

[68] Archer J.W., Dobbs M.R., Aydin A., Reeves H.J., Prance R.J. (2016). Measurement and correlation of acoustic emissions and pressure stimulated voltages in rock using an electric potential sensor. Int. J. Rock Mech. Min. Sci..

[69] Freund F. (2011). Pre-earthquake signals: Underlying physical processes. J. Asian Earth Sci..

[70] Freund F.T. (2007). Pre-earthquake signals—Part I: Deviatoric stresses turn rocks into a source of electric currents. Nat. Hazards Earth Syst. Sci..

[71] Freund F.T. (2007). Pre-earthquake signals—Part II: Flow of battery currents in the crust. Nat. Hazards Earth Syst. Sci..

[72] Takeuchi A., Aydan Ö., Sayanagi K., Nagao T. (2011). Generation of electromotive force in igneous rocks subjected to non-uniform loading. Earthq. Sci..

[73] Theimer B.D., Nobes D.C., Warner B.G. (1994). A study of the geoelectrical properties of peatlands and their influence on ground-penetrating radar surveying1. Geophys. Prospect..

[74] Jouniaux L., Pozzi J.-P. (1995). Permeability dependence of streaming potential in rocks for various fluid conductivities. Geophys. Res. Lett..

[75] Knight R.J., Nur A. (1987). The dielectric constant of sandstones, 60 kHz to 4 MHz. Geophysics.

[76] Niu Q., Zhang C., Prasad M. (2020). A Framework for Pore-Scale Simulation of Effective Electrical Conductivity and Permittivity of Porous Media in the Frequency Range from 1 mHz to 1 GHz. J. Geophys. Res. Solid Earth.

[77] Porretta R., Bianchi F. (2016). Profiles of Relative Permittivity and Electrical Conductivity from Unsaturated Soil Water Content Models. Ann. Geophys..

[78] Cartwright-Taylor A., Vallianatos F., Sammonds P. (2014). Superstatistical view of stress-induced electric current fluctuations in rocks. Phys. A Stat. Mech. Its Appl..

[79] Chen H.-J., Ye Z.-K., Chiu C.-Y., Telesca L., Chen C.-C., Chang W.-L. (2020). Self-Potential Ambient Noise and Spectral Relationship with Urbanization, Seismicity, and Strain Rate Revealed via the Taiwan Geoelectric Monitoring Network. J. Geophys. Res. Solid Earth.

[80] Fisher R.A. (1925). Theory of Statistical Estimation. Math. Proc. Camb. Philos. Soc..

[81] Shannon C.E. (1948). A Mathematical Theory of Communication. Bell Syst. Tech. J..

[82] Vignat C., Bercher J.-F. (2003). Analysis of signals in the Fisher–Shannon information plane. Phys. Lett. A.

[83] Frieden B.R. (1990). Fisher information, disorder, and the equilibrium distributions of physics. Phys. Rev. A.

[84] Frieden B.R., Soffer B.H. (1995). Lagrangians of physics and the game of Fisher-information transfer. Phys. Rev. E.

[85] Martin M., Perez J., Plastino A. (2001). Fisher information and nonlinear dynamics. Phys. A Stat. Mech. Its Appl..

[86] Sen K.D., Antolín J., Angulo J.C. (2007). Fisher-Shannon analysis of ionization processes and isoelectronic series. Phys. Rev. A.

[87] Baravalle R., Rosso O.A., Montani F. (2018). Causal Shannon–Fisher Characterization of Motor/Imagery Movements in EEG. Entropy.

[88] Telesca L., Lovallo M. (2011). Analysis of the time dynamics in wind records by means of multifractal detrended fluctuation analysis and the Fisher–Shannon information plane. J. Stat. Mech. Theory Exp..

[89] Telesca L., Lovallo M., Hsu H.-L., Chen C.-C. (2011). Analysis of dynamics in magnetotelluric data by using the Fisher–Shannon method. Phys. A Stat. Mech. Its Appl..

[90] Telesca L., Lovallo M. (2013). Fisher-Shannon Analysis of Wind Records. Int. J. Energy Stat..

[91] Telesca L., Lovallo M., Chamoli A., Dimri V.P., Srivastava K. (2013). Fisher–Shannon analysis of seismograms of tsunamigenic and non-tsunamigenic earthquakes. Phys. A Stat. Mech. Its Appl..

[92] Telesca L., Lovallo M., Romano G., Konstantinou K.I., Hsu H.-L., Chen C.-C. (2014). Using the informational Fisher–Shannon method to investigate the influence of long-term deformation processes on geoelectrical signals: An example from the Taiwan orogeny. Phys. A Stat. Mech. Its Appl..

[93] Telesca L., Lovallo M., Ramírez-Rojas A., Angulo-Brown F. (2009). A nonlinear strategy to reveal seismic precursory signatures in earthquake-related self-potential signals. Phys. A Stat. Mech. Its Appl..

[94] Telesca L., Lovallo M., Carniel R. (2010). Time-dependent Fisher Information Measure of volcanic tremor before the 5 April 2003 paroxysm at Stromboli volcano, Italy. J. Volcanol. Geotherm. Res..

[95] Potirakis S.M., Minadakis G., Eftaxias K. (2012). Analysis of electromagnetic pre-seismic emissions using Fisher information and Tsallis entropy. Phys. A Stat. Mech. Its Appl..

[96] Potirakis S.M., Minadakis G., Nomicos C.D., Eftaxias K. (2011). A multidisciplinary analysis for traces of the last state of earthquake generation in preseismic electromagnetic emissions. Nat. Hazards Earth Syst. Sci..

[97] Telesca L., Lapenna V., Lovallo M. (2005). Fisher Information Analysis of earthquake-related geoelectrical signals. Nat. Hazards Earth Syst. Sci..

[98] Esquivel R.O., Angulo J.C., Antolín J., Dehesa J.S., López-Rosa S., Flores-Gallegos N. (2010). Analysis of complexity measures and information planes of selected molecules in position and momentum spaces. Phys. Chem. Chem. Phys..

[99] Romera E., Dehesa J.S. (2004). The Fisher–Shannon information plane, an electron correlation tool. J. Chem. Phys..

[100] Angulo J.C., Antolín J., Sen K.D. (2008). Fisher–Shannon plane and statistical complexity of atoms. Phys. Lett. A.

[101] Janicki A., Weron A. (1993). Simulation and Chaotic Behavior of Alpha-Stable Stochastic Processes.

[102] Devroye L. (1987). A Course in Density Estimation.

[103] Troudi M., Alimi A.M., Saoudi S. (2008). Analytical Plug-in Method for Kernel Density Estimator Applied to Genetic Neutrality Study. Eurasip J. Adv. Signal Process..

[104] Raykar V.C., Duraiswami R. (2006). Fast optimal bandwidth selection for kernel density estimation. Proceedings of the 2006 SIAM International Conference on Data Mining.

[105] Telesca L., Lovallo M. (2017). On the performance of Fisher Information Measure and Shannon entropy estimators. Phys. A Stat. Mech. Its Appl..

[106] Potirakis S.M., Karadimitrakis A., Eftaxias K. (2013). Natural time analysis of critical phenomena: The case of pre-fracture electromagnetic emissions. Chaos Interdiscip. J. Nonlinear Sci..

[107] Ramirezrojas A., Telesca L., Angulo-Brown F. (2011). Entropy of geoelectrical time series in the natural time domain. Nat. Hazards Earth Syst. Sci..

[108] Varotsos P.A., Skordas E., Sarlis N., Lazaridou M.S. (2008). Fluctuations, under time reversal, of the natural time and the entropy distinguish similar looking electric signals of different dynamics. J. Appl. Phys..

[109] Varotsos P.A., Sarlis N.V., Skordas E.S. (2014). Study of the temporal correlations in the magnitude time series before major earthquakes in Japan. J. Geophys. Res. Space Phys..

[110] Sarlis N.V., Skordas E.S., Varotsos P.A. (2010). Order parameter fluctuations of seismicity in natural time before and after mainshocks. EPL Europhys. Lett..

